# Unilateral optic disc drusen mis-diagnosed as optic neuritis: Diagnostic and therapeutic implications

**DOI:** 10.22336/rjo.2020.69

**Published:** 2020

**Authors:** Georgios Bontzos, Georgios Smoustopoulos, Theocharis Efstathios Detorakis

**Affiliations:** * Department of Ophthalmology, Medical School, University of Crete, Heraklion, Greece

**Keywords:** optic-disc, drusen, pseudo-papilledema

## Abstract

**Objective:** To present a case of unilateral optic disc drusen, initially mis-diagnosed as optic neuritis, which led to chronic systemic administration of steroids and the development of hypercortisolism.

**Methods:** A 22-year-old female was referred because of the lack of improvement of the manifestations of optic neuritis despite the chronic use of systemic steroids. Presence of unilateral optic disc edema was initially observed, associated with ipsilateral scotomata and increased ipsilateral latency time in visually evoked potentials (VEP).

**Results:** A CT scan, A and B ultrasonography and autofluorescence of the optic disc confirmed the diagnosis of optic disc drusen.

**Conclusions:** In cases of optic disc edema, the possibility of drusen should always be examined even if functional disturbances, such as scotomas in visual fields or electrophysiological findings, are present, to avoid potential toxicity from unnecessary medications.

## Introduction

Optic disc drusen are acellular calcifications of the optic disc, either superficial or deep, occurring in up to 2% of the general population [**1**]. The lesions are bilateral in 75% of the cases and may increase in size in aging individuals [**1**]. Usually they are asymptomatic, however, they may occasionally cause vascular disorders, such as haemorrhages, retinal vein occlusion or venous stasis retinopathy [**2**,**3**]. It should be noted that the most significant clinical implication of optic disc drusen is the fact that they might be confused with true optic disc edema [**4**]. We presented a case of young woman with unilateral optic disc drusen associated with ipsilateral visual field defects and increased ipsilateral latency time in visually evoked potentials (VEP), who was mis-diagnosed as suffering from optic neuritis for which she received large amounts of steroids with considerable systemic morbidity.

## Case Report

**Fig. 1:**
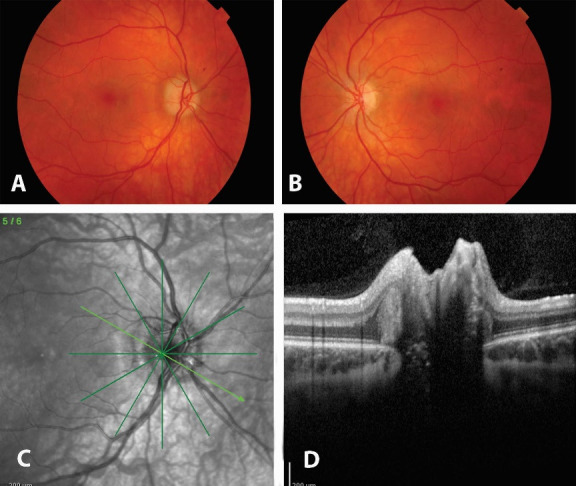
Fundus color photos of right (A) and left (B) eyes, showing irregular fluffy borders of the optic disc of the right eye. OCT scan (C,D) of the optic disc area of the right eye, shows elevation of the optic disc

A 22-year-old female was referred to the Department of Ophthalmology of the University Hospital of Heraklion due to persistent edema of the right optic disc (**Fig. 1A**). Previous systemic and ophthalmic histories were non-contributory. The edema of the right optic disc was discovered during a routine ophthalmic examination for glasses prescription and was confirmed by an OCT examination (**Fig. 1C, D**). 

**Fig. 2:**
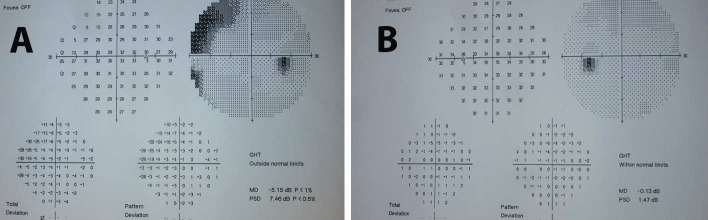
Visual fields (30°) of the right (A) and left (B) eyes, showing a scotoma at the superior-nasal quadrant of the visual fields of the right eye

**Fig. 3:**
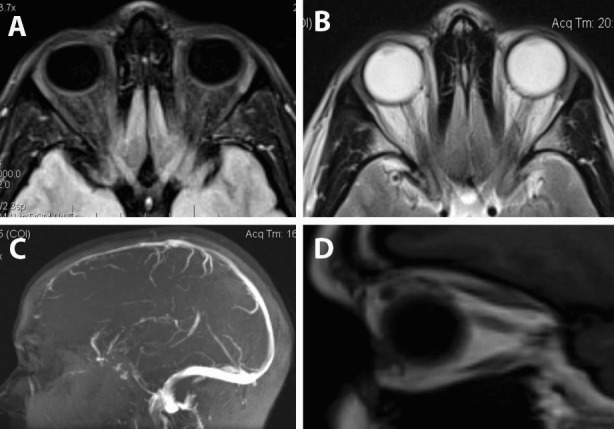
MRI scans of the orbits of the patient. T1-oriented contrast enhanced and fat suppressed scan (A), T-2 oriented scan (B), both at transverse planes. T1-oriented sagittal plane scan (D). MR angiography of the brain (C). All scans are non-contributory concerning orbital or intracranial pathology

**Fig. 4:**
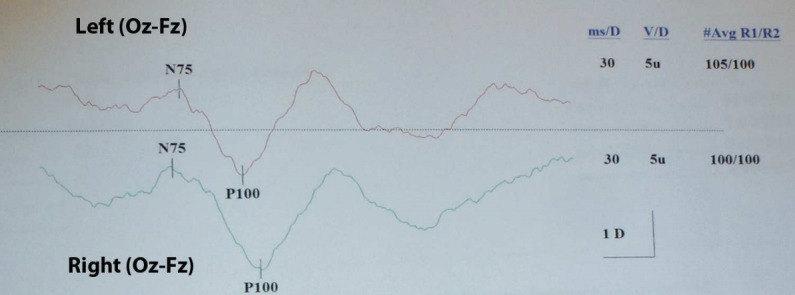
Output of checkerboard VEP of both eyes showing an increased latency time for the right eye

Subsequently, the patient underwent visual field testing, which was significant for scotomata at the visual field of the right eye (**Fig. 2**). Accordingly, her ophthalmologist referred her to a neurologist for further evaluation. Taking into account the presence of unilateral optic disc edema with associated visual field defects and her young age, the possibility of optic neuritis was raised and the patient underwent MRI and MR angiography of the brain (**Fig. 3**), which were however, non-contributory. The patient then underwent checkerboard VEP, which confirmed an increased latency time interval for the right eye, compared with the left eye (**Fig. 4A**). At that point, the diagnosis of optic neuritis of unspecified origin was considered highly likely and the patient was administered systemic steroids for 3 months in a tapering dosage. However, steroid treatment did not improve visual field findings. Moreover, the optic disc edema persisted and the patient was referred to the Department of Ophthalmology for further management.

**Fig. 5:**
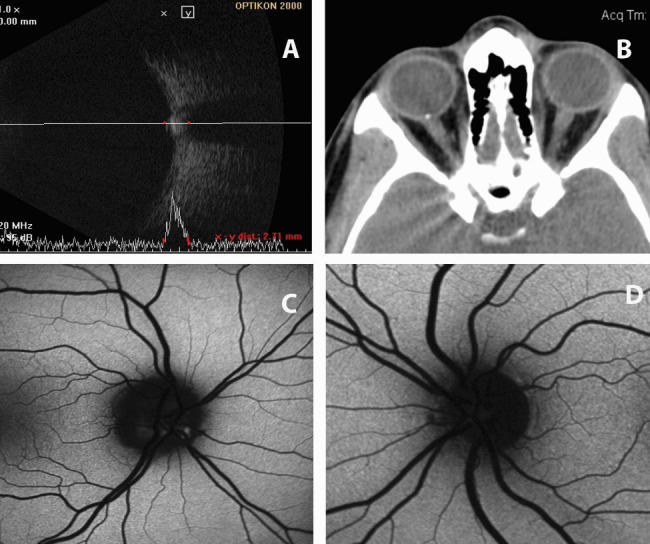
A and B ultrasound scans of the right eye showing a highly reflective area at the optic nerve head, compatible with drusen (A). CT scan of the orbits showing a bone density area at the optic nerve head of the right eye (B). Auto-fluorescence fundoscopic images of the right (C) and left (D) eyes, showing fluorescent bodies at the optic nerve head of the right eye, compatible with drusen

Upon presentation, facial edema was noted, compatible with steroid-related Cushing’s syndrome. Best corrected visual acuity (BCVA) was 20/ 20 OU, color perception was normal and the IOP was 18 mmHg (OD) and 17 mmHg (OS) without medications. Slit lamp biomicroscopy was non-contributory. Fundoscopy was indeed significant for optic disc edema (OD) with normal appearance of the optic disc for the OS. However, considering the lack of changes in the appearance of the optic disc, following administration of steroids and the fact that BCVA or color vision were not compromised, the suspicion of optic disc drusen was raised. Accordingly, B-scan and A-scan ultrasound evaluations were performed (**Fig. 5A**), which confirmed the presence of a highly reflective area at the optic disc of the right eye. Moreover, CT imaging (**Fig. 5B**) and auto-fluorescence (**Fig. 5C,D**) were significant for the presence of a fluorescent body and high density area, respectively, on the right optic disc. Therefore, the presence of the optic disc drusen was confirmed.

## Discussion

Optic disc drusen often present as an incidental finding in routine clinical practice [**5**]. The problem of the differential diagnosis of optic disc drusen from true optic disc edema is more complicated in children, due to the fact that in this age group optic disc drusen may occasionally co-exist with intracranial hypertension [**6**,**7**]. Moreover, optic disc drusen have been associated with various clinical ophthalmic findings, such as abnormal vascular branching, cilioretinal vessels, peripapillary atrophy and haemorrhages [**8**]. Visual fields defects, relative afferent pupillary defects and an increased latency time in multi-focal VEP have also been associated with optic disc drusen in previous reports [**9**,**10**]. Such changes may be associated with compression on the optic nerve fibers, caused by the accumulation of calcified deposits in a tight space, such as the optic nerve head [**10**]. 

In the case presented, the diagnostic uncertainties associated with the finding of a unilateral optic disc edema and the obvious concern of physicians who initially treated the patient not to miss an important diagnosis, such as an episode of optic neuritis, led to the administration of systemic steroids, which in turn resulted in serious side-effects (the development of Cushing’s syndrome). This is also the reason why additional diagnostic modalities, such as VEPs or MR angiographic studies, were employed to evaluate this condition. Ironically, the presence of optic disc drusen would have been obvious if simpler diagnostic methods, such as A and B ultrasound scans or CT scans were initially employed. Therefore, primary care providers, either ophthalmologists or optometrists, should bear in mind that optic disc elevation or blurred margins, even when associated with findings such as visual field defects, could be the result of optic disc drusen and accordingly employ adequate available diagnostic modalities (such as ultrasound and CT scans) before referring the patient to tertiary facilities for further management. 

## Conclusion

Optic disc drusen are a well-known cause of pseudo-papilledema. Although, typically asymptomatic, common manifestations include peripheral scotomas that are abnormal pupillary reflexes. Suspicion of this entity is raised by careful clinical examination and patient’s history. Diagnosis can be confirmed by autofluorescence or ultrasonography.

**Conflict of Interest**

The authors state no conflict of interest.

**Informed Consent**

An informed consent was obtained from the patient included in this Case Report.

**Authorization for the use of human subjects**

The research related to human use complies with all the relevant national regulations, institutional policies, is in accordance with the tenets of the Helsinki Declaration, and has been approved by the Ethics Committee of the Department of Ophthalmology of the University Hospital of Heraklion. 

**Acknowledgements**

None.

**Sources of Funding**

None.

**Disclosures**

None.
